# Likely Response to a Hypothetical Menthol Cigarette Ban Among Adults with Mood Disorders Who Smoke Menthol Cigarettes and Have No Current Plans to Quit Smoking

**DOI:** 10.3390/ijerph21111477

**Published:** 2024-11-06

**Authors:** Laraib Mazhar, Jonathan Foulds, Sophia I. Allen, Susan Veldheer, Shari Hrabovsky, Jessica M. Yingst

**Affiliations:** 1 Department of Public Health Sciences, Penn State College of Medicine, Hershey, PA 17033, USA; jfoulds@pennstatehealth.psu.edu (J.F.); sallen3@pennstatehealth.psu.edu (S.I.A.); sveldheer@pennstatehealth.psu.edu (S.V.); jyingst@pennstatehealth.psu.edu (J.M.Y.); 2 Department of Family and Community Medicine, Penn State College of Medicine, Hershey, PA 17033, USA; 3 Penn State Nese College of Nursing, University Park, PA 16802, USA; shrabovsky@pennstatehealth.psu.edu

**Keywords:** menthol cigarettes, mood disorders, smoking behavior, addiction, tobacco additives

## Abstract

Background: There is limited evidence on how the United States Food and Drug Administration’s (FDA) proposed ban on menthol cigarettes and flavored cigars will impact individuals with mood disorders who smoke menthol cigarettes. This study aimed to evaluate how individuals with mood disorders who smoke menthol cigarettes might respond to a hypothetical ban on menthol cigarettes, explore the reasons for their current use, and examine how these reasons are associated with participants’ characteristics. Methods: Study data were collected at baseline from adults (18+ years) with mood disorders who participated in a randomized controlled trial evaluating the impact of gradual nicotine reduction. Participants were individuals who smoked and had no plan to quit in the next six months. They reported demographics and tobacco consumption patterns, interest in quitting, and responded to a hypothetical question on a potential ban on menthol cigarettes. The question asked participants which actions they would most likely take if menthol-flavored cigarettes were banned. Means and frequencies were used to describe the sample. Logistic regression was used to determine factors associated with each reason for menthol use (less harmful, better flavor, less harsh on the throat, and less harsh on the chest). Results: Participants (*n* = 77) were an average age of 42.5 (SD 12.5) years, 61% (*n* = 47) were female, 68.8% (*n* = 53) identified as White, and 5.2% (*n* = 4) identified as Hispanic. On average, participants reported currently smoking 18.1 (SD 9.9) cigarettes per day and had smoked for 23.9 (SD 13.6) years. About 58.4% of participants (*n* = 45) expressed their intention to switch to non-menthol cigarettes, 19.5% (*n* = 15) intended to transition to a different type of tobacco product, and 22.1% (*n* = 17) intended to quit smoking entirely without substitution. The most endorsed reason for using menthol cigarettes was better flavor (89.6%, *n* = 69/77), followed by less harshness on the throat (41.3%, *n* = 31/75) and chest (40%, n = 30/75), and the belief that they were less harmful than non-menthol cigarettes (24%, *n* = 18/75). Older age was associated with the belief that menthol cigarettes were less harmful (OR = 1.06; *p* = 0.02). Conclusion: Among individuals with mood disorders and who smoke menthol cigarettes and have no plans to quit smoking, 22.1% may try to quit smoking if a menthol ban is implemented, while the majority (58.4%) stated that they would switch to non-menthol cigarettes. As the reasons for using menthol cigarettes included perceived lower harm, there is a need for targeted public awareness campaigns to correct misconceptions about the harms of menthol cigarettes.

## 1. Introduction

The addition of menthol in cigarettes creates a minty taste and cooling sensation in the throat and chest, making smoking smoother and less harsh, resulting in a deeper inhalation of tobacco smoke [1]. Individuals who smoke often prefer menthol cigarettes for several reasons, including the mistaken belief that they are less harmful, as well as the cooling sensation, enhanced flavor, and gentler effect on the throat and lungs [2,3,4]. Some studies have found that individuals who smoke menthol cigarettes tend to have greater nicotine dependence, resulting in greater difficulty in attempting to quit cigarette smoking [5,6]. In the United States, the prevalence of menthol cigarette use among adults who smoke has significantly increased from 33.8% in 2008 to 40.6% in 2019 [7]. Over 80% of Black individuals aged 18–34 years old who currently smoke cigarettes use menthol cigarettes, which is the highest rate across racial and ethnic groups [7]. Menthol cigarette use exacerbates health disparities among Black or African American individuals, Hispanic individuals, women, and those with lower incomes, which is largely due to targeted marketing and limited access to cessation resources [8,9,10]. In addition, adults with mental health conditions are found to be more likely to smoke menthol cigarettes than those without such conditions, further highlighting the intersection of menthol use and vulnerable populations. [11,12].

In April 2022, the United States Food and Drug Administration (FDA) proposed product standards to ban menthol as a characterizing flavor in cigarettes and eliminate all non-tobacco characterizing flavors in cigars. This initiative aims to reduce the harms associated with menthol cigarettes and protect at-risk groups from initiating and continuing tobacco use [13]. Thus, restricting the sale of menthol cigarettes has the potential to contribute to the reduction in the burden of tobacco use among groups disproportionately affected [14].

To understand the implications of a ban on menthol cigarettes, several studies have examined individuals who smoked menthol cigarettes’ hypothetical responses to such a ban. A scoping review found that 11–45% of US adults who smoke menthol cigarettes would quit in response to a menthol ban, while 15–30% would consider switching to e-cigarettes as a substitute [15]. One potential reason for interest in switching to electronic cigarettes is the availability of flavors, including menthol [16]. A study evaluating a Canadian menthol ban estimated that a similar ban in the US could lead to quitting cigarettes among more than 1.3 million individuals who smoke [17].

While previous research [3,18,19] has examined reactions to a potential menthol ban among various subgroups of the population, there is little evidence on the likely effects of a ban on menthol cigarettes among people with mood disorders who smoke. This group is known to smoke menthol cigarettes at a higher rate. Understanding the effects of the menthol ban on individuals with mood disorders who smoke is important to promote equitable access to healthcare. Therefore, this study aimed to assess the likely response to a hypothetical ban on menthol cigarettes among people with mood disorders who smoke and had no plans to quit smoking in the next 6 months, determining whether they would switch to non-menthol cigarettes, switch to another product, or quit smoking entirely. Additionally, we explored the reasons for their current menthol cigarette use and examined how these reasons are associated with participants’ characteristics. Findings from this study may guide the development of tailored public health strategies and regulatory measures aimed at reducing tobacco use among this population group.

## 2. Methods

Participants were adults (18 years or older) with mood disorders who smoked menthol cigarettes and were enrolled in a randomized controlled trial on gradual nicotine reduction in cigarettes, reporting no quit attempts in the past month and no plans to quit in the next six months. In short, participants were current individuals who smoke menthol cigarettes, consuming at least 5 cigarettes per day, and met the criteria for a current or lifetime unipolar mood disorder (such as dysthymia, major or minor depression, premenstrual dysphoric disorder) or anxiety disorder (such as panic disorder, obsessive-compulsive disorder, post-traumatic stress disorder, mixed anxiety–depressive disorder, agoraphobia, generalized anxiety disorder, social phobia, specific phobia) based on the structured Mini-International Neuropsychiatric Interview (MINI) [20] and must not have an unstable medical or psychiatric condition [21]. Additionally, they had not used varenicline, bupropion (used specifically as a quitting aid), nicotine patch, gum, lozenge, inhaler, or nasal spray in the prior month. At baseline, participants self-reported their intention to quit based on their response to the screener statement: ‘I have no plans to quit or significantly cut back within the next 6 months’, which described their current situation. Participants for the study were recruited from Pennsylvania and Massachusetts, with the study being conducted at the Penn State College of Medicine in Hershey, PA, and Massachusetts General Hospital in Boston, MA. Full details and results from this study can be found here [21,22,23].

This report focuses on data that were collected from participants at the baseline visit of the study. Out of the 188 participants in the main trial, 77 individuals smoked menthol cigarettes and were included in this study. Participants reported basic demographic information, including age, gender, education level, income, race, and ethnicity. Also, they reported cigarettes per day and the total years of smoking. Participants were asked about their response to a hypothetical ban on menthol cigarettes. Specifically, they were asked to indicate their most likely course of action in response to the question, “If menthol cigarettes were no longer sold, which of the following would you most likely do? (1. Switch to non-menthol cigarettes, 2. Switch to some other tobacco product, 3. Quit smoking and not use any other tobacco product).” Reasons for menthol use were assessed with the following question, “For each of the following, please tell us whether it’s a reason you usually smoke menthol cigarettes… (1) They are less harmful than non-menthol, (2) They have a better flavor than non-menthol, (3) They are less harsh on your throat than non-menthol, (4) They are less harsh on your chest than non-menthol (Yes/No)”. Each potential predictor variable (age, gender, education, race, ethnicity, and household income) was individually assessed with the outcome.

### Statistical Analysis

Means and frequencies were used to describe the sample and outcomes. A binary variable was created to assess interest in quitting, and responses were grouped into two categories: those indicating a switch to non-menthol cigarettes or another tobacco product were categorized as “no”, and responses indicating quitting were categorized as a “yes”. We conducted a multicollinearity assessment before performing univariate analysis, and none of the independent variables were correlated. Unadjusted analyses were first conducted using univariate logistic regression to identify variables associated with each outcome, using a *p*-value threshold of ≤0.25. Variables that met this threshold were included in an adjusted multivariable logistic regression model with significance set at *p* ≤ 0.05. Stepwise logistic regression with forward selection was applied to determine factors associated with interest in quitting menthol cigarettes if a ban were imposed as well as with each reason for menthol use (i.e., less harmful, better flavor, less harsh on the throat, and less harsh on the chest). In the adjusted models, we controlled for demographic factors such as age, gender, education level, income, race, and ethnicity.

## 3. Results

Participants (*n* = 77) were an average age of 42.5 (SD 12.5) years, 61% (*n* = 47) were female, 68.8% (*n* = 53) identified their race as White, and 5.2% (*n* = 4) identified as Hispanic. On average, participants reported smoking 18.1 (SD 9.9) cigarettes per day and had been smoking for an average of 23.9 (SD 13.6) years. Few participants had obtained a college degree (10.4%, *n* = 8), and they had an annual household income greater than $50,000 (20.8%, *n* = 16). The most prevalent mood disorders reported by the participants were major depressive disorder (76.6%, *n* = 59), agoraphobia (49.4, *n* = 38), and panic disorder (44.2%, *n* = 34), as shown in Table 1. We identified that 62.3% (*n* = 48) had two or more MINI mood/anxiety disorder diagnoses, highlighting a large portion of the sample with multiple conditions.

**Figure 1:**
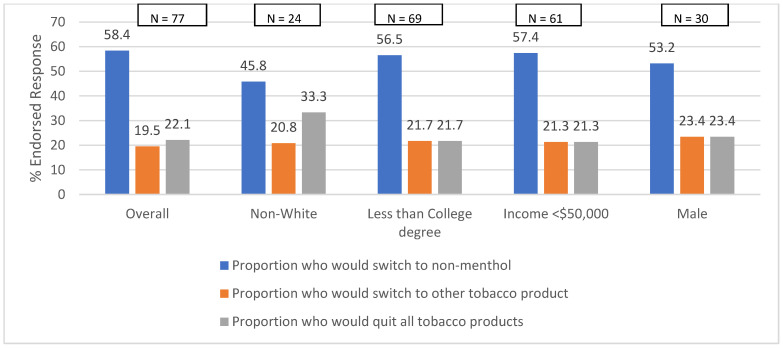
Response to hypothetical ban of menthol cigarettes.

In response to a hypothetical ban on menthol cigarettes, 58.4% of participants (*n* = 45) expressed their intention to switch to non-menthol cigarettes, while 19.5% (*n* = 15) stated that they would transition to a different type of tobacco product. Over one fifth (22.1%, *n* = 17) reported that they would quit smoking entirely without substituting with any other tobacco products (Figure 1).

When interest in quitting was treated as a binary variable, we found that the interest in quitting was associated with Hispanic ethnicity (*p* = 0.03) and White race (*p* = 0.11) at the univariate level, with both variables meeting the cutoff of *p* ≤ 0.25 for inclusion in the final multivariable model. In the multivariable analysis, no variables (including Hispanic ethnicity and White race) showed a statistically significant association with the interest in quitting, as shown in Table 2.

The most endorsed reason (89.6%, *n* = 69) for using menthol cigarettes was “better flavor than non-menthol cigarettes”. More than one-third of participants said they used menthol because it was less harsh on their throat (41.3%, *n* = 31/75) and chest (40%, *n* = 30/75). About one quarter (24%, *n* = 18/75) used menthol cigarettes because they believed they were less harmful than non-menthol. In the logistic regression models for menthol use, the only reason with a significant predictor was “menthol cigarettes are less harmful”. We found that older age was associated with believing menthol cigarettes are less harmful (OR= 1.06; *p* = 0.03). No other predictors were significant, as shown in Table 2.

## 4. Discussion

Findings from our study shed light on the potential outcomes of a menthol cigarette ban among individuals with mood disorders who smoke menthol cigarettes. Our study suggested that a significant proportion of individuals with mood disorders who smoke menthol cigarettes and have no plans to quit in the next 6 months may continue smoking by switching to non-menthol tobacco products. The Canadian menthol ban showed that a menthol flavor ban could disrupt tobacco use patterns and encourage quitting among individuals who smoke menthol cigarettes [17]. While our study cannot be compared with the previous studies, our findings indicate a tendency for individuals with mood disorders who smoke menthol cigarettes to switch to non-menthol cigarettes instead. Countries like Canada, the UK, and the Netherlands found that banning menthol as an additive is more effective than banning it solely as a characterizing flavor [24]. A comprehensive ban on menthol, as both an additive and a characterizing flavor, may significantly reduce menthol smoking prevalence in the United States.

Only 22.1% of participants in this study reported an intention to stop smoking completely. However, it is important to note that these findings cannot be directly compared to estimates from larger, national samples, which have reported intentions to quit ranging from 25% to 64%, as seen in the scoping review exploring the impact of the menthol cigarette ban on individual behaviors [15]. A key distinction in our study is that participants were selected based on having ‘no plans to quit in the next 6 months’, which significantly limits the comparability of our findings to those of national studies where the participants may not have had such criteria. Further, participants in this study had mental health conditions that might have affected their responses to a hypothetical menthol ban.

While quitting all tobacco use is ideal for health, for those unwilling or unable to quit all tobacco, the use of less harmful alternative tobacco products could be a viable option [25]. We found that nearly 20% of participants indicated they would switch to other tobacco products, indicating a potential shift in tobacco use behaviors possibly influenced by regulatory measures. Kotlyar et al. found that menthol smokers who abstained from cigarettes used e-cigarettes more frequently, indicating that e-cigarettes may serve as a useful alternative when menthol cigarettes are unavailable [26]. Future studies should focus on understanding the potential alternatives chosen by individuals who smoke. Such studies are crucial for assessing the overall impact of a menthol ban.

It is important to note that about one-quarter of participants smoked menthol cigarettes because of a perception that menthol cigarettes are less harmful than non-menthol. Previous studies have reported that African American individuals, young adults, and women consider menthol cigarettes as less harmful [27]; however, in our study, the only predictor of this belief was older age, indicating a potential generational difference in harm perceptions of menthol cigarette smoking. The findings also suggest that beliefs about the relative harm of menthol cigarettes, especially among older adults, may need to be addressed in public health campaigns to correct the misconceptions and promote quitting behaviors.

This study has several limitations. A significant limitation of this study is the small sample size. Individuals with mood disorders who smoke and have no plans to quit smoking in the next 6 months may display distinct smoking behaviors, motivations, and responses to interventions compared to the general population. Various mental health conditions can uniquely influence smoking behaviors and motivations. Treating these diverse conditions as a single group may limit the accuracy of the findings. The findings should be interpreted cautiously because of the small sample size and inclusion of participants with no plans to quit. Future research with larger sample sizes should include subgroups of specific mood disorders to provide more accurate insights. Another limitation is the lack of information regarding the specific product and flavor to which individuals who smoke would switch if menthol cigarettes were banned. In addition, we did not ask about other ways of obtaining menthol cigarettes, including the black market or making your own menthol cigarettes. Moreover, the study was conducted in specific geographical locations (Central PA and Boston, MA, USA). Therefore, the results may not be representative of the broader population of individuals who smoke and have mood disorders. It is important to interpret the study findings cautiously, especially when applying them to the general population. Individuals who smoke and with these disorders may respond differently to a menthol ban compared to those without. Although 58% of participants in this study indicated they would switch to non-mentholated cigarettes, it is important to consider that the psychological and behavioral characteristics of this group may influence their responses in ways that are not representative of the general smoking population. Future studies should include comprehensive inquiries in diverse and representative samples. These studies should consider factors such as past experiences, preferences, cost, availability, perceived health risks, nicotine dependence, and motivation to quit.

## 5. Conclusions

Our findings suggest that if a menthol ban is passed in the US, less than one-quarter of individuals with mood disorders who smoke menthol cigarettes and have no plans to quit may choose to quit smoking. However, a significant proportion may opt for tobacco alternatives instead of quitting altogether. The findings also suggest the importance of beliefs about the relative harm of menthol cigarettes, especially among older adults. Public health awareness campaigns should correct misconceptions and promote quitting behaviors.

## Figures and Tables

**Table 1:** Participant characteristics.

Characteristics	Sample (*n* = 77)
Sociodemographic	
Age, mean (SD, range)	42.5 (12.5, 21–64)
Female, %(n)	61.0 (47)
White, %(n)	68.8 (53)
Hispanic, %(n)	5.2 (4)
Earned college degree, %(n)	10.4 (8)
Income > $50,000, %(n)	20.8 (16)
Smoking history	
Cigarettes per day, %(n)	18.1 (9.9)
Total Years Smoking, %(n)	23.9 (13.6)
Mental health conditions	
Major depressive disorder, %(n)	76.6 (59)
Minor depressive disorder, %(n)	7.8 (6)
Dysthymia, %(n)	7.8 (6)
Manic episode, %(n)	3.9 (3)
Hypomanic episode, %(n)	1.3 (1)
Panic disorder, %(n)	44.2 (34)
Agoraphobia, %(n)	49.4 (38)
Social phobia, %(n)	7.8 (6)
Obsessive–compulsive disorder, %(n)	7.8 (6)
Post-traumatic stress disorder, %(n)	36.4 (28)
Generalized anxiety disorder, %(n)	22.1 (17)
Attention-deficit/hyperactivity disorder, %(n)	18.2 (14)
Premenstrual dysphoric disorder, %(n)	9.1 (7)
Number of mood disorder diagnoses	
One diagnosis %(n)	29 (37.7)
Two or more diagnoses %(n)	48 (62.3)

**Table 2:** Logistic regression analysis of factors influencing interest in quitting and reasons for menthol cigarette use.

Predictor/Outcome	Interest in Quitting if Menthol Cigarettes Were Banned	Reason for Menthol Use: Menthol Cigarettes Are Less Harmful	Reason for Menthol Use: Menthol Cigarettes Have Better Flavor	Reason for Menthol Use: Menthol Cigarettes Are Less Harsh on Throat	Reason for Menthol Use: Menthol Cigarettes Are Less Harsh on Chest
	Unadjusted OR (95% CI, *p*-Value)	Unadjusted OR (95% CI, *p*-Value)	Unadjusted OR (95% CI, *p*-Value)	Unadjusted OR (95% CI, *p*-Value)	Unadjusted OR (95% CI, *p*-Value)
Age	0.99 (0.96–1.04, 0.98)	1.06 (1.01–1.12; 0.03) *	1.02 (0.96, 1.09; 0.53)	1.01 (0.97–1.06; 0.54)	1.02 (0.97–1.06; 0.50)
Male (Ref: Female)	0.82 (0.27–2.51; 0.73)	1.49 (0.47–4.73; 0.50)	1.44 (0.29, 7.15; 0.65)	2.34 (0.83–6.59; 0.11)	2.0 (0.69–5.80; 0.20)
White (Ref: Non-White)	0.40 (0.14–1.24; 0.11)	0.75 (0.22–2.51; 0.64)	2.82 (0.55, 14.43; 0.21)	0.56 (0.18–1.71; 0.31)	0.37 (0.12–1.16; 0.09)
Hispanic (Ref: Non-Hispanic)	12.64 (1.22–130.85; 0.03)	1.01 (0.08–12.48; 0.99)	**	**	**
Earned college degree (Ref: Earned less than a college degree)	1.2 (0.22–6.57; 0.83)	1.57 (0.30–8.24; 0.59)	0.80 (0.08, 8.44)	0.81 (0.16–4.05; 0.79)	0.45 (0.08–2.66; 0.38)
Income > $50,000 (Ref: Income < $50,000)	1.23 (0.34–4.56; 0.75)	0.45 (0.08–2.38; 0.34)	**	0.8 (0.23–2.82; 0.73)	0.85 (0.24–3.07; 0.80)

* In the multivariable analysis examining the reason that “menthol cigarettes are less harmful”, age was the only significant predictor after adjusting for gender, education, race, ethnicity, and household income (odds ratio = 1.06; *p* = 0.03). None of the other models had significant predictors at the multivariable level. ** Due to sparse data, odds ratios for this variable could not be calculated.

## Data Availability

The data underlying this article will be shared on reasonable request to the corresponding author.
